# Supplementary material for: Complement Depletion Improves Human Red Blood Cell Reconstitution in Immunodeficient Mice
Source: Stem Cell Reports. 2017 Sep 28;9(4):1034–42. doi: 10.1016/j.stemcr.2017.08.018 (PMC5639386; doi:10.1016/j.stemcr.2017.08.018)
Supplement: Document S1. Supplemental Experimental Procedures and Figures S1–S4 [file mmc1.pdf]

Stem Cell Reports, Volume *9*

Supplemental Information

Complement Depletion Improves Human Red Blood Cell Reconstitution in Immunodeficient Mice

Bing Chen, Wei Fan, Jun Zou, Siwen Zhang, Jin He, Chang Shu, Guoqing Zhao, Tianmeng Sun, Zheng Hu, and Yong-Guang Yang

Supplemental Information

Complement depletion improves human red blood cell reconstitution in immunodeficient mice

Bing Chen, Wei Fan, Jun Zou, Siwen Zhang, Jin He, Chang Shu, Guoqing Zhao, Tianmeng Sun, Zheng Hu, Yong-Guang Yang

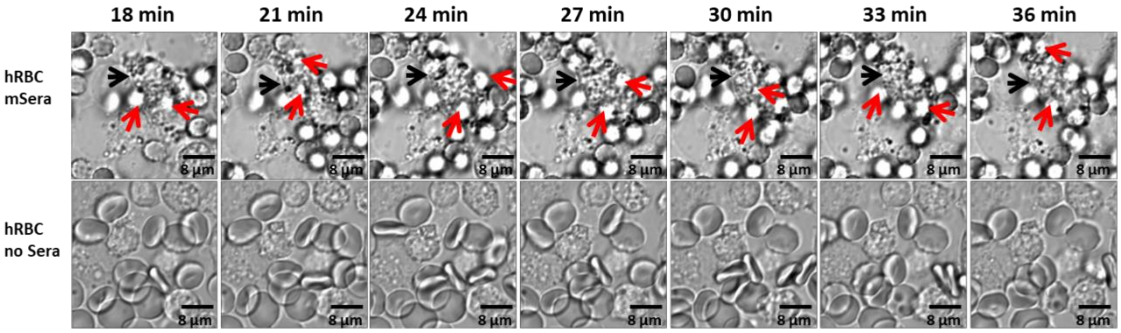

Figure S1. Human RBC adherence to and engulfment by mouse phagocytic cells.
Human RBCs were co-cultured with NOD/SCID mouse peritoneal cells in the presence of 15% NOD/SCID mouse sera (top) or absence of sera (bottom) at 37 °C in Delta Vision Imaging System (the same experiment shown in Movie S1 and S2). Shown are representative imagines captured at indicated time points. In the cultures with mouse sera (top), human RBCs and mouse phagocytic cells are distinguished by red and black arrows, respectively. Scale bars represent 8 μm.

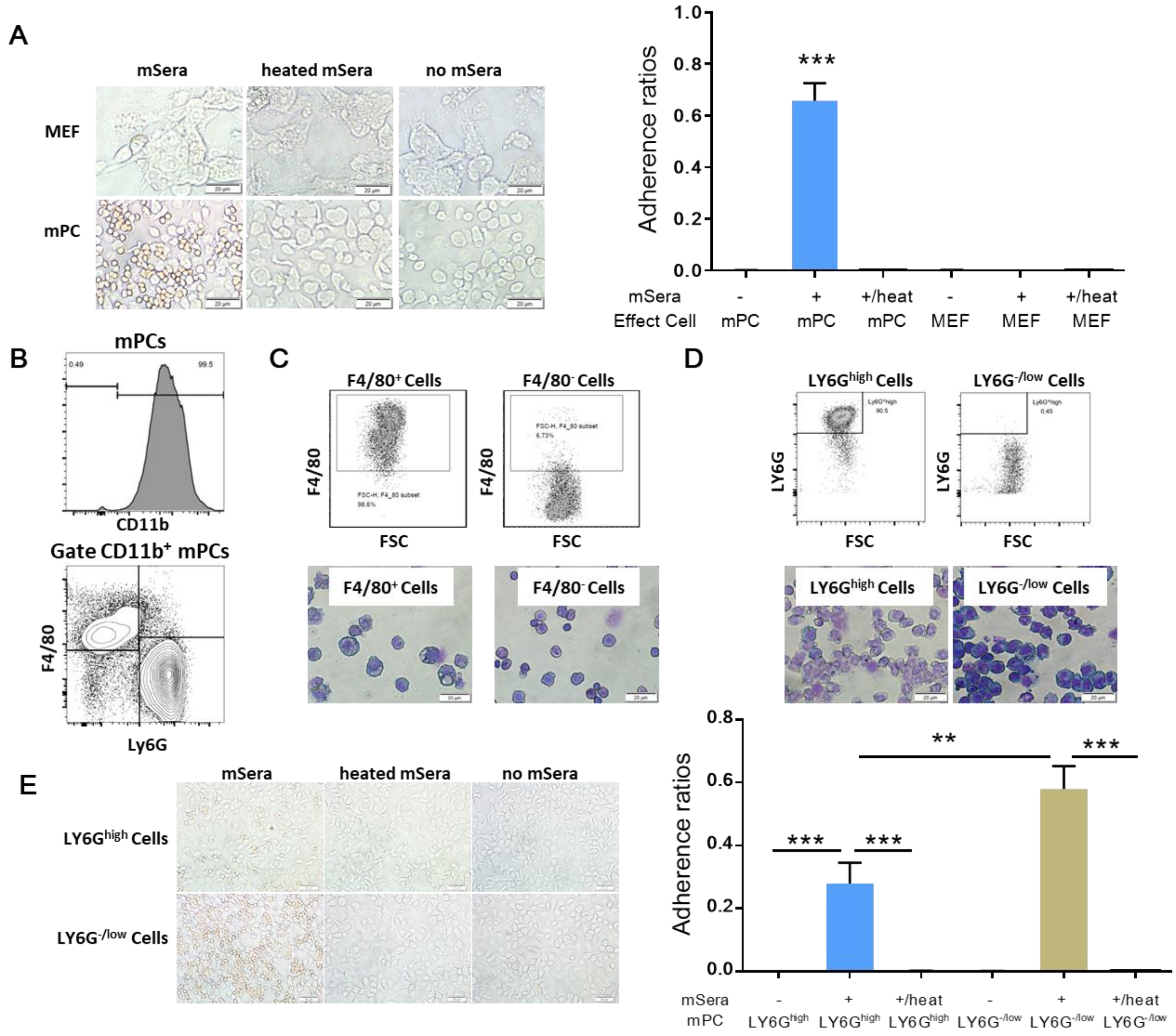

Figure S2. Adherence of human RBCs to mouse phagocytic and non-phagocytic cells.
(A) Adherence of human RBCs (hRBCs) to NOD/SCID mouse peritoneal cells (mPCs) or mouse embryonic fibroblast (MEF) cells in the presence or absence of NOD/SCID mouse sera (mSera). Representative pictures (left) and adherence ratios (right; mean±SDs; n=4 technical replicates) are shown. ***, p<0.001. (B) Flow cytometric analysis of CD11b, F4/80 and Ly6G expression on mPCs. (C, D) F4/80$^+$ and F4/80$^-$ (C) or LY6G$^+$ and LY6G$^-$ (D) mPCs were isolated by magnetic-activated cell sorting, and stained with Wright-Giemsa staining after cytospin. Flow cytometry profiles showing the purity of the sorted cells (top) and representative images of Wright-Giemsa staining (bottom) are shown. (E) Adherence of hRBCs to purified LY6G$^+$ and LY6G$^-$ mPCs in the presence of untreated or heated NOD/SCID mouse sera (mSera), or in serum-free medium. Representative pictures (left) and adherence ratios (right; mean±SDs; n=4 technical replicates) are shown. Scale bars represent 20 μm. **, p<0.01; ***, p<0.001.

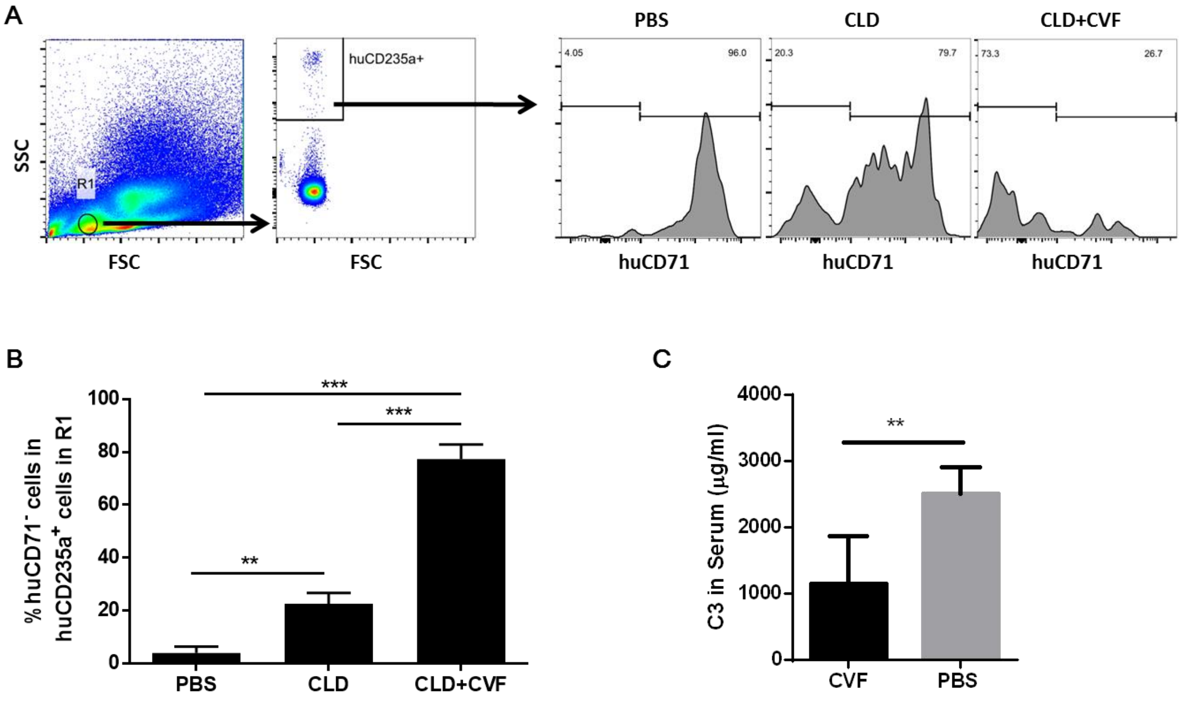

Figure S3. CVF treatment improves human erythropoiesis in human CD34^{+} cell-grafted NOD/SCID mice that are depleted of macrophages.

NOD/SCID mice were irradiated and injected with 3×10^{5} CD34^{+} cells, and treated 4 weeks later with PBS (n=3 mice per group), clodronate-liposomes (CLD; 3 injections; 100 µL at day 0, 25 µL at day 2 and day 5; n=3 mice per group), or CLD and CVF (3 injections; 5 µg at day 0, 2, 5; n=3 mice per group). Animals were sacrificed 7 days later and analyzed for human CD71^{-}CD235a^{+} erythroid cells in bone marrow by flow cytometry. (A) Representative staining profiles of human CD71 expression on gated human CD235a^{+} cells in the "mature erythrocyte–enriched" gate (R1). (B) Percentages (mean±SDs) of CD71^{-}CD235a^{+} erythroid cells in R1 gate. (C) Levels of mouse serum C3 in CVF-treated (n=3 mice per group) and PBS-injected (n=6 mice per group) mice at day 7 after treatment. **, p<0.01; ***, p<0.001.

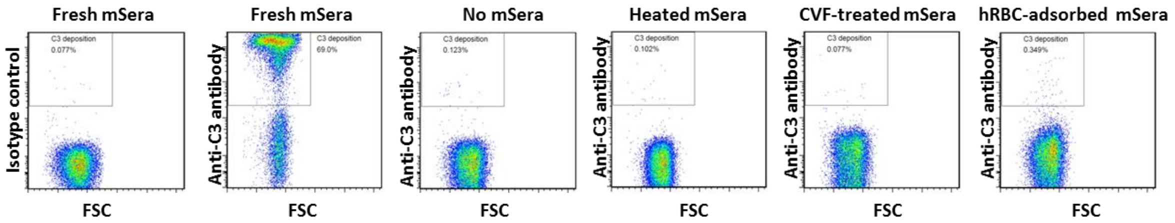

Figure S4. Mouse complement deposition on human RBCs opsonized with mouse sera. Human RBCs were incubated in fresh, heated, CVF treated, or human RBC-adsorbed mouse sera (mSera), or in serum-free medium for 30 minutes, and analyzed for C3 deposition by flow cytometry. Representative flow cytometric profiles from a representative of 2 independent experiments are shown.

Supplemental Movies

Movie S1. Human RBCs and mouse PCs cultured in the presence of 15% NOD/SCID mouse sera.
Movie S2. Human RBCs and mouse PCs cultured in absence of mouse sera.

Supplemental Experimental Procedures

Morphologic analyses of mouse peritoneal cavity cells from NOD/SCID mice

$F4/80^+$ and $F4/80^-$ or $LY6G^+$ and $LY6G^-$ mouse PCs were purified from NOD/SCID mouse peritoneal cavity 4 days after Bio-Gel i.p. injection, suspended in PBS, and centrifuged (130g for 5 minutes) onto glass slides using a Cytospin centrifuge (Shandon). The slides were stained with Wright Giemsa staining kit (Leagene). Stained slides were examined under an Olympus microscope and photographed using an Olympus DP73 digital color camera.

ELISA

Concentration of mouse complement C3 in mouse sera was analyzed by ELISA kit specific for mouse complement C3 (ab157711, Abcam), according to the manufacture's instructions.

Complement deposition assay

Human RBCs were incubated for 30 minutes in serum-free medium, or with fresh, heated, CVF-treated (at 5μg CVF per 300 μL sera), or human RBC-adsorbed (incubated with hRBCs at 5×10^9 cells per 300 μL sera) NOD/SCID mouse sera. After incubation, the cells were washed twice and stained with rat anti-mouse complement C3 mAb (CL7503, CEDARLANE) or rat IgG_{2a} isotype control antibody, and analyzed by flow cytometry.
