# Supplementary figures and images for: Complement Depletion Improves Human Red Blood Cell Reconstitution in Immunodeficient Mice
Source: Stem Cell Reports. 2017 Sep 28;9(4):1034–42. doi: 10.1016/j.stemcr.2017.08.018 (PMC5639386; doi:10.1016/j.stemcr.2017.08.018)

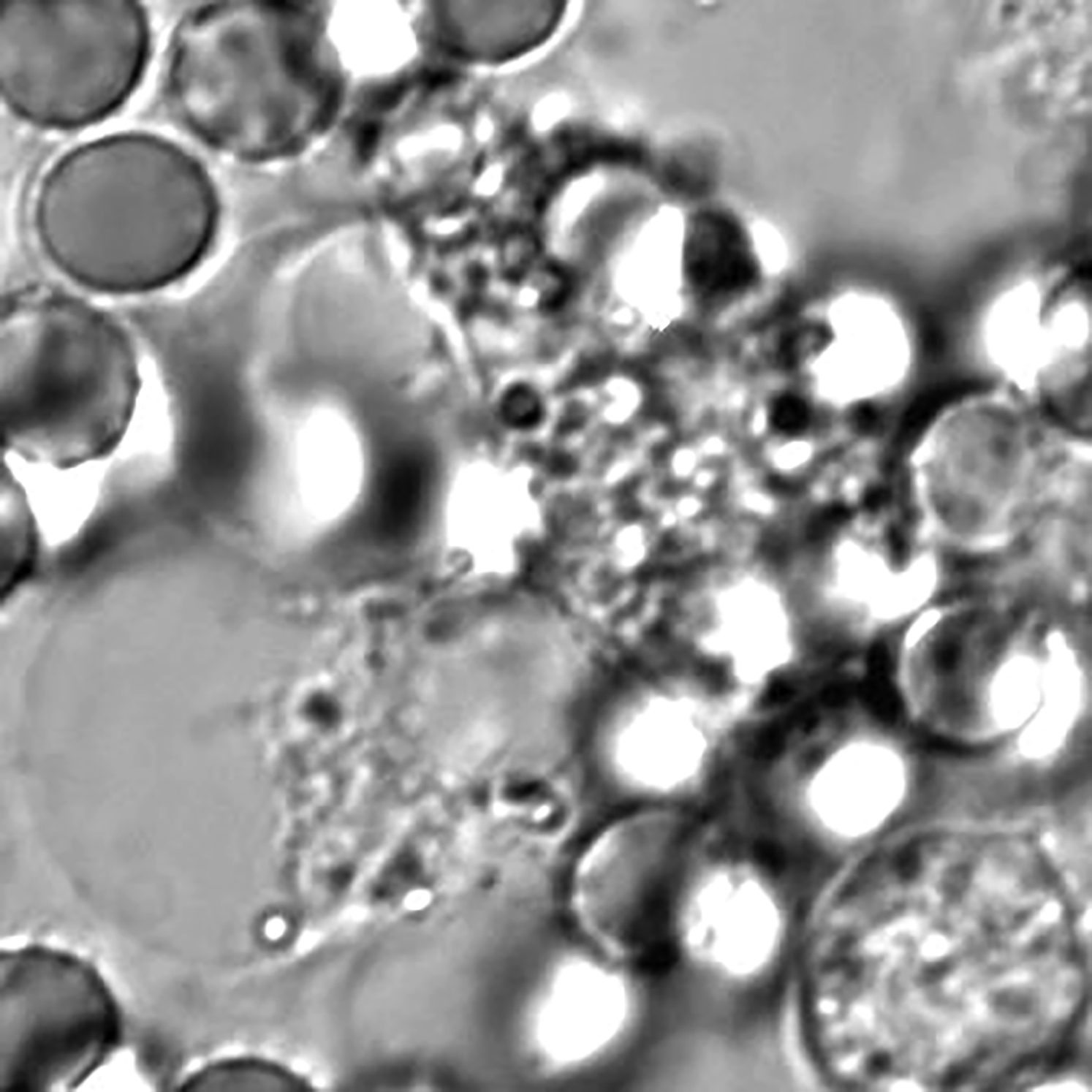

Supplement: Movie S1. Human RBCs and Mouse PCs Cultured in the Presence of 15% NOD/SCID Mouse Sera [file mmc2.jpg]

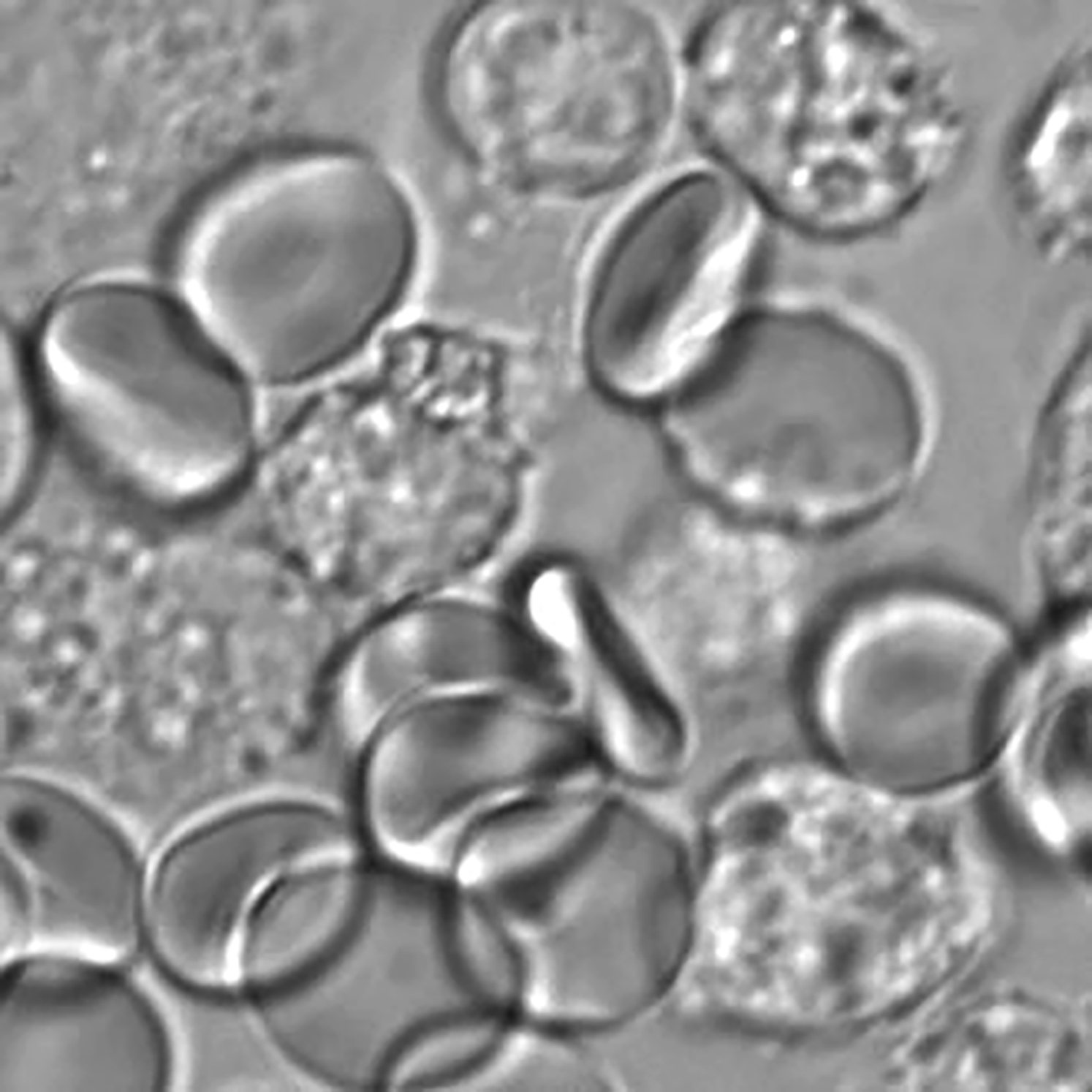

Supplement: Movie S2. Human RBCs and Mouse PCs Cultured in Absence of Mouse Sera [file mmc3.jpg]
