# Supplementary material for: Complement Depletion Improves Human Red Blood Cell Reconstitution in Immunodeficient Mice
Source: Stem Cell Reports. 2017 Sep 28;9(4):1034–42. doi: 10.1016/j.stemcr.2017.08.018 (PMC5639386; doi:10.1016/j.stemcr.2017.08.018)
Supplement: Document S2. Article plus Supplemental Information [file mmc4.pdf]

Stem Cell Reports
Report

ISSCR

OPEN ACCESS

Complement Depletion Improves Human Red Blood Cell Reconstitution in Immunodeficient Mice

Bing Chen,[1,2,3,4] Wei Fan,[1,2,3] Jun Zou,[1,2,3] Siwen Zhang,[1] Jin He,[1] Chang Shu,[1] Guoqing Zhao,[4] Tianmeng Sun,[1,2,3] Zheng Hu,[1,2,3,*] and Yong-Guang Yang[1,2,3,5,*]
[1]The First Bethune Hospital and Institute of Immunology, Jilin University, Changchun 130061, China
[2]National-Local Joint Engineering Laboratory of Animal Models for Human Diseases, Changchun 130061, China
[3]International Center of Future Science, Jilin University, Changchun 130012, China
[4]China-Japan Union Hospital of Jilin University, Changchun 130033, China
[5]Columbia Center for Translational Immunology, Department of Medicine, Columbia University College of Physicians and Surgeons, New York 10032, USA
*Correspondence: zhenghu0108@outlook.com (Z.H.), yy2324@columbia.edu (Y.-G.Y.)
http://dx.doi.org/10.1016/j.stemcr.2017.08.018

SUMMARY

We have previously shown that human red blood cells (hRBCs) are subject to robust rejection by macrophages in immunodeficient mice. In this study, we found that mouse serum induces hRBC adherence to murine phagocytic cells, including professional phagocytic macrophages and neutrophils and non-professional phagocytic endothelial cells. Complement was found to be responsible for mouse-serum-induced hRBC adherence to murine phagocytic cells. Although hRBC survival was not improved in NOD/SCID mice with complement depletion by cobra venom factor (CVF), CVF significantly prolonged hRBC survival in mice that were depleted of phagocytic macrophages by clodronate-liposomes. This combination treatment also synergistically improved hRBC reconstitution in human CD34^{+} cell-grafted mice, offering a valuable model to examine human erythropoiesis and RBC function. These data indicate that complement, which might be dispensable for hRBC rejection by macrophages, is critical in hRBC rejection by other types of murine phagocytic cells, such as neutrophils and endothelial cells.

INTRODUCTION

As one of the most plentiful cell types in body, the red blood cell (RBC) is indispensable in oxygen and carbon dioxide transport (Goodnough et al., 2000). In adults, RBCs differentiate from a limited number of hematopoietic stem cells (HSCs) in bone marrow in a process called erythropoiesis, which is a complicated and tightly regulated program that comprises distinct erythroid progenitor stages (Palis, 2014). Any genetic error in this process may lead to hematological diseases, such as β-thalassemia and hereditary spherocytosis (Da Costa et al., 2013). The recent development of effective gene editing strategies, such as CRISPR/Cas9 technology (Suzuki et al., 2016), has increased the potential of curing these hematological disorders through transplantation of patient HSCs with the genetic errors corrected (Rees et al., 2010; Sankaran and Weiss, 2015). However, these studies have been hampered by the lack of a suitable animal model that permits *in vivo* assessment of human erythroid differentiation from adult HSCs or induced pluripotent stem cell (iPSC)-derived HSCs and RBC function (Sankaran and Weiss, 2015).

Immunodeficient mice have been used widely for human HSC transplantation (Hu and Yang, 2012a). Although human HSC engraftment leads to the differentiation of multiple lineages of human hematopoietic cells, human RBC (hRBC) reconstitution cannot be achieved in these mice following human HSC transplantation, primarily due to rejection by murine phagocytic cells (Hu et al., 2011; Hu and Yang, 2012b). Although fully matured CD71^{-}CD235a^{+} enucleated hRBCs can be detected in humanized mice after macrophage depletion by clodronate-liposome injection, their levels are insufficient, limiting its value as an *in vivo* model for the study of human hematological disorders, malaria infection, and relevant therapeutic interventions (Hu et al., 2011). The low levels of hRBCs in human HSC-grafted mice that have been depleted of macrophages imply that other macrophage-independent mechanisms are involved, necessitating optimization of humanized mice with stable and high levels of hRBC chimerism in blood (Rahmig et al., 2016; Rongvaux et al., 2013).

In this study, we found that mouse complement is critical in mediating the rejection of hRBCs in immunodeficient mice. We show that elimination of murine complement by cobra venom factor (CVF) nearly completely abrogated the adherence of hRBCs to murine phagocytic cells *in vitro* and that CVF significantly prolonged the survival of infused hRBCs in macrophage-depleted mice. Moreover, combining CVF with macrophage depletion increased hRBC reconstitution in human CD34^{+} cell-grafted mice, constituting a valuable pre-clinical model to examine the efficacy and safety of RBC differentiation from gene-edited human HSCs.

This is an open access article under the CC BY license (http://creativecommons.org/licenses/by/4.0/).

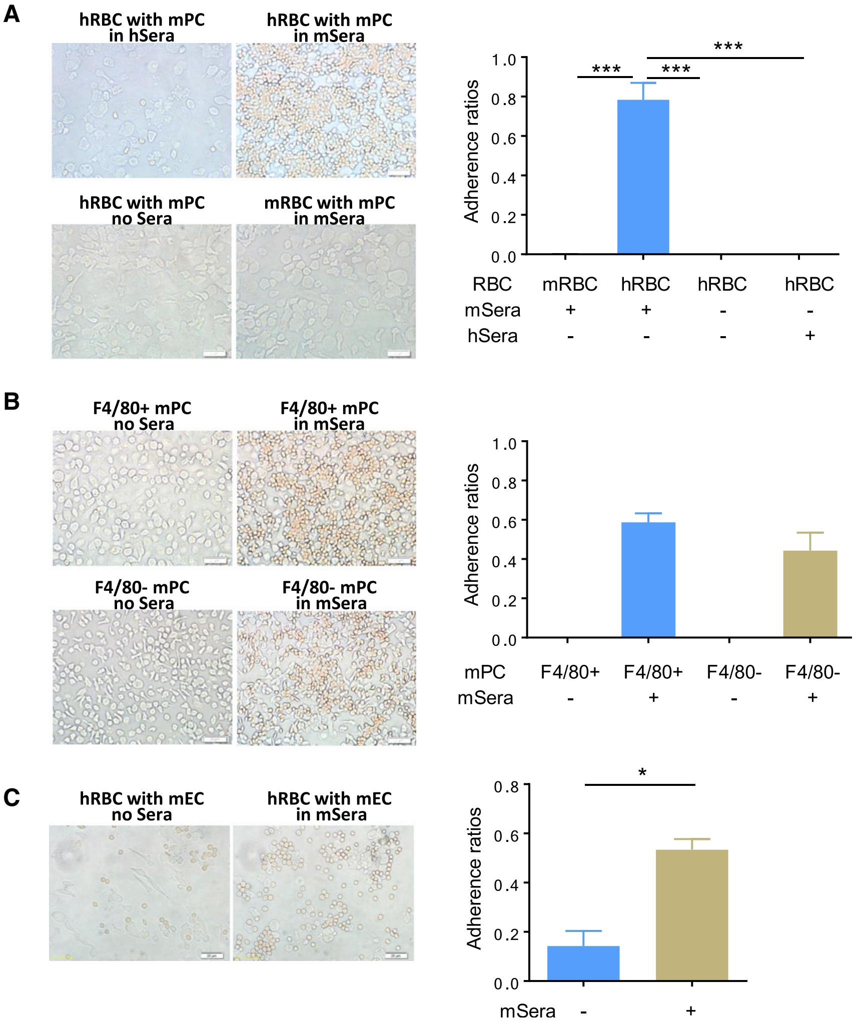

Figure 1. Adherence of Human RBCs on Mouse Myeloid Phagocytic Cells and Endothelial Cells in the Presence of Mouse Sera

(A) Adherence of human RBCs (hRBCs) or mouse RBCs (mRBCs) to NOD/SCID mouse peritoneal cells (mPCs) in the presence of human sera (hSera) or NOD/SCID mouse sera (mSera), or in serum-free medium. Representative pictures (left) and adherence ratios (right; mean ± SD; n = 3 independent experiments) are shown.

(B) Adherence of hRBCs to purified F4/80$^+$ and F4/80$^-$ NOD/SCID mPCs. Representative pictures (left) and adherence ratios (right; mean ± SD; n = 4 technical replicates from a representative of 2 independent experiments) are shown.

(C) Adherence of hRBCs to mouse CD31$^+$ endothelial cells (mECs). Representative pictures (left) and adherence ratios (right; mean ± SD; n = 3 independent experiments) are shown.

Scale bars represent 20 μm. *$p < 0.05$; ***$p < 0.001$; n.s., not significant.

RESULTS

Mouse, but Not Human, Sera Promote the Adherence of Human RBCs to Murine Phagocytic Cells

Because adherence to phagocytic cells is a significant event in the phagocytosis of target cells, we first assessed the potential of mouse sera to induce adherence of hRBCs to murine phagocytic cells. Human RBCs adhered to non-obese diabetic/severe combined immunodeficiency (NOD/SCID) mouse peritoneal cells (PCs) in the presence of NOD/SCID mouse sera but not human sera or in serum-free medium (Figures 1A and S1; Movies S1 and S2). However, mouse sera did not induce mouse RBC adherence to mouse PCs (Figure 1A) or hRBC adherence to mouse non-phagocytic fibroblast cells (Figure S2A). Flow cytometric and cytospin analysis revealed that the majority of PCs from NOD/SCID mice were CD11b$^+$ myeloid cells that consisted mainly of F4/80$^+$Ly6G$^-$ macrophages and F4/80$^-$Ly6G$^+$ neutrophils (Figures S2B–S2D). Human RBCs were found to adhere to both F4/80$^+$ (or LY6G$^-$) macrophages and F4/80$^-$ (or LY6G$^+$) neutrophils, to a greater extent to the former, in the presence of mouse sera (Figures 1B and S2E). Endothelial cells (ECs) are non-professional phagocytic cells that clear apoptotic cells (Dini et al., 1995; Graham et al., 2009; Yu et al., 2016). Thus, we next measured hRBC adherence to CD31$^+$ mouse ECs in the presence and absence of mouse sera. As shown in Figure 1C, NOD/SCID mouse sera also elicited significant hRBC adherence to mouse ECs. These data demonstrate the ability of mouse sera to induce hRBC adherence to murine phagocytic cells, including macrophages and neutrophils, and non-professional phagocytic ECs. The adherence of hRBCs to mouse neutrophils and ECs in the presence of mouse sera explains our previous observation that, despite a significant improvement in hRBC survival, the rejection of hRBCs in NSG mice cannot be prevented

completely by treatment with macrophage-depleting clodronate-liposomes (Hu et al., 2011).

Opsonization of Human RBCs by Mouse Complement Causes Human RBC Adherence to Murine Phagocytic Cells

To determine whether mouse-serum-induced adherence of hRBCs to murine phagocytic cells was due to its effect on hRBCs, mouse phagocytic cells, or both, we measured the impact of pretreatment of hRBCs or mouse PCs with mouse sera on their adherence. Cell adherence was not detected when hRBCs were cultured in serum-free medium with untreated or mouse-serum-treated PCs (Figure 2A). In contrast, mouse-serum-treated hRBCs adhered significantly to untreated mouse PCs when cultured in serum-free medium, to a level comparable with the adherence between untreated hRBCs and untreated PCs that were cultured in mouse sera (Figure 2A). The data indicate that the adherence of hRBCs to mouse PCs or the recognition of hRBCs by murine phagocytic cells in the presence of mouse sera is induced by mouse-serum-induced opsonization of hRBCs. In support of this possibility, we observed that hRBC-pre-adsorbed mouse sera (i.e., mouse sera that had been incubated with a saturating amount of hRBCs) did not cause the adherence of hRBCs to mouse PCs (Figure 2B). Thus, opsonization of hRBCs by mouse serum opsonins is likely to be the major mechanism for mouse-serum-induced adherence of hRBCs to mouse PCs.

To characterize the mouse serum opsonins that induce hRBC adherence to mouse PCs, we evaluated the potential of preheated (at 56°C for 30 min) mouse sera to induce hRBC adherence to mouse phagocytic cells in comparison with untreated mouse sera. Unlike in untreated mouse sera, hRBCs did not show significant adherence to mouse PCs or ECs when cultured in preheated mouse sera (Figure 2C). These data indicate that the mouse serum opsonins that promote adherence of hRBCs to mouse phagocytic cells are heat labile.

Mice on the NOD background lack hemolytic complement C5 (Baxter and Cooke, 1993), but produce opsonic complement components, such as C3b (Patel and Harrison, 2008). Because complement proteins are heat labile (Hair et al., 2012; Lewis et al., 2008; Nauta et al., 2004; Patel and Harrison, 2008), it is possible that complement is the major serum opsonin that induces hRBC adherence to mouse phagocytic cells. In support of this possibility, we found that mouse sera treated with CVF, a complement-activating component of cobra venom that activates and thus exhausts C3 and C5 (Gowda et al., 1994), did not induce hRBC adherence to mouse PCs (Figure 2D). Further, sera prepared from CVF-treated NOD/SCID mice also failed to induce adherence of hRBCs to mouse PCs (Figure 2D). Consistent with these observations, the addition of mouse complement proteins induced hRBC adherence to mouse PCs dose dependently in cultures without mouse sera or with preheated NOD/SCID sera (Figure 2E).

CVF Treatment Prolongs the Survival of Human RBCs in Macrophage-Depleted Immunodeficient Mice

We examined the potential of complement depletion to inhibit hRBC rejection in NOD/SCID mice. Human RBC survival was not improved in NOD/SCID mice that were treated with CVF compared with PBS-injected controls (Figure 3A), despite the fact that the sera from the former failed to induce hRBC adherence to murine phagocytic cells *in vitro* (Figure 2D). In contrast, the same dose of CVF significantly increased hRBC survival (by approximately 3-fold) in NOD/SCID mice that were depleted of macrophages by clodronate-liposome injection (Figure 3B). Further, multiple injections of CVF prolonged the survival of infused hRBCs (up to approximately 3 weeks) in macrophage-depleted NOD/SCID mice (Figure 3C).

CVF Significantly Improves Human RBC Reconstitution in Human HSC-Grafted Immunodeficient Mice with Macrophage Depletion

NOD/SCID B2m mice received 1.5 Gy total body irradiation, followed by intravenous injection of human $CD34^+$ cells. Flow cytometric analysis of blood cells at week 7 post-transplantation revealed significant reconstitution with human $CD45^+$ cells (ranging from 6.81% to 48.9% of peripheral blood mononuclear cells) but no detectable hRBCs (Figure 4A). At week 8 post-transplantation, these mice were treated with clodronate-liposomes, CVF, clodronate-liposomes and CVF, or PBS (as controls), and monitored for hRBC chimerism in blood. Consistent with the hRBC transfusion experiments (Figure 3), hRBCs were only detected in mice that were depleted of macrophages (Figure 4B). In these mice, hRBCs appeared by day 5 and continued to increase throughout the observation period of 16 days, and the levels of hRBC chimerism in mice that were treated with both clodronate-liposomes and CVF were approximately 2-fold higher than in those treated with clodronate-liposomes alone (Figure 4B). In contrast, hRBCs remained nearly undetectable in CVF-treated mice and PBS-injected controls (Figure 4B). We have previously shown that macrophage depletion by clodronate-liposomes results in a significant increase in mature erythroid ($CD71^-CD235a^+$) cells in bone marrow (Hu et al., 2011). Compared with mice treated with clodronate-liposomes, a further increase in $CD71^-CD235a^+$ erythroid cells were detected in the bone marrow from mice treated with a combination of clodronate-liposomes and CVF (Figures S3A and S3B). These results suggest a synergistic effect of clodronate-liposomes and CVF on the inhibition of hRBC rejection in immunodeficient mice.

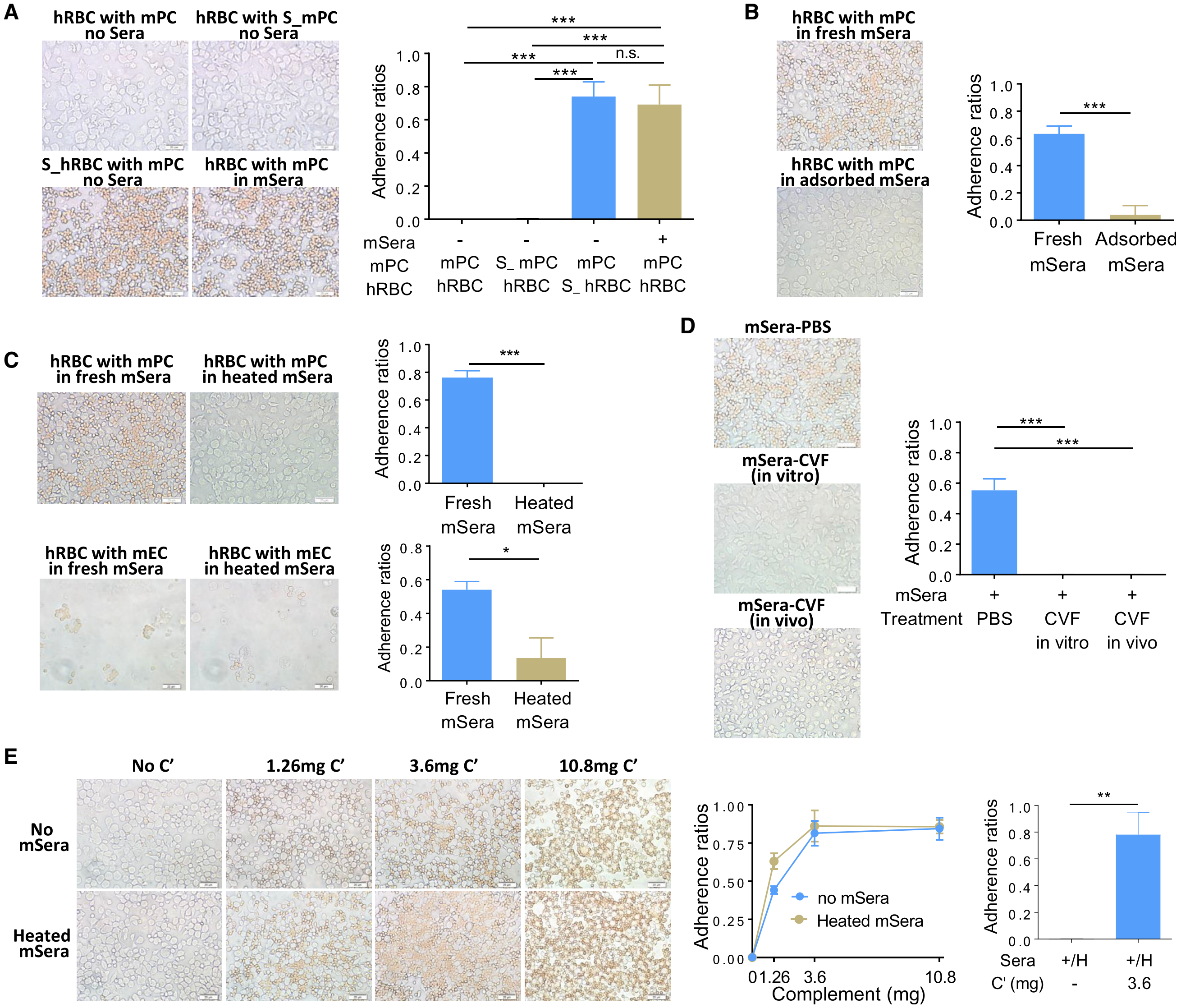

Figure 2. Mouse Complement Opsonization Induces Adherence of Human RBCs to Mouse Phagocytic Cells

(A) Adherence of untreated hRBCs or mouse serum (mSerum)-pretreated hRBCs (S_hRBC) to untreated mouse PCs (mPCs) or mSerum-treated mPCs (S_mPCs). Left, representative pictures; right, adherence ratios (mean ± SD, n = 3 independent experiments).

(B) Adherence of hRBCs to mPCs in the presence of fresh mSera or mSera that were adsorbed with hRBCs (2.5×10^9 hRBCs for 100 μL mSera). Left, representative pictures; right, adherence ratios (mean ± SD, n = 3 independent experiments).

(C) Adherence of hRBCs to mPCs (top panel) or mouse ECs (mECs; bottom panel) in the presence of fresh or heated mSera. Left, representative pictures; right, adherence ratios (mean ± SD, n = 3 independent experiments).

(D) Adherence of hRBCs to mPCs in the presence of NOD/SCID mSera treated with PBS (mSera-PBS) or CVF (mSera-CVF [*in vitro*]), or mSera prepared from CVF-treated NOD/SCID mice (mSera-CVF [*in vivo*]). Left, representative pictures; right, adherence ratios (mean ± SD, n = 3 independent experiments).

(E) Adherence between hRBCs and mPCs co-cultured in mSera-free medium or heated mSera with or without addition of mouse complement proteins. Left, representative pictures; middle, adherence ratios in mSera-free medium or heated mSera with the indicated amounts of mouse complement proteins (n = 4 technical replicates); right, adherence ratios in heated mSera with addition of 3.6 mg mouse complement proteins (mean ± SD, n = 3 independent experiments).

Scale bars represent 20 μm. $^*p < 0.05$; $^{**}p < 0.01$; $^{***}p < 0.001$; n.s., not significant.

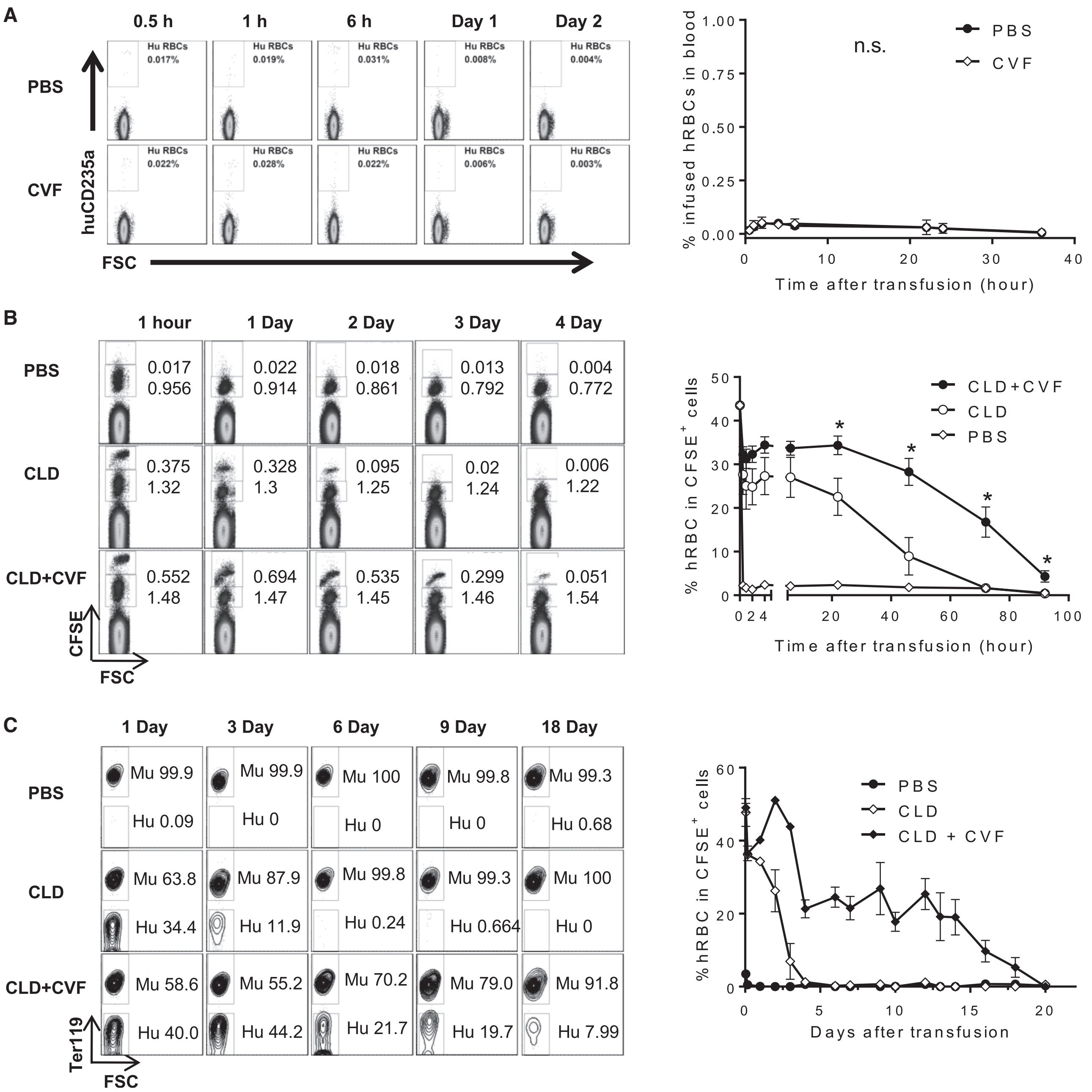

Figure 3. CVF Treatment Prolongs RBC Survival in Immunodeficient Mice with Macrophage Depletion

(A) CFSE-labeled hRBCs were intravenously injected into NOD/SCID mice that were treated with PBS (n = 3 mice per group) or CVF (10 μg at day −1 and 5 μg at day 0; n = 5 mice per group). Blood was collected at the indicated times and analyzed for hRBC chimerism by flow cytometry. Representative flow cytometric profiles (left) and summarized data (right; mean ± SD) were shown.

(B) Human (hRBCs) and NOD/SCID mouse (mRBCs) RBCs were labeled with high and low intensity of CFSE, respectively, mixed at approximately 1:1 ratio, and infused into NOD/SCID mice that were treated with PBS-liposomes (PBS; n = 3 mice per group) or clodronate-liposomes (CLD, 100 μL at day −1, 50 μL at day 0; n = 3 mice per group) or clodronate-liposomes plus CVF (CLD + CVF; 100 μL CLD and 10 μg CVF at day −1; 50 μL CLD and 5 μg CVF at day 0; n = 4 mice per group). Blood was collected at the indicated times and the levels of surviving hRBCs and mRBCs were assessed by flow cytometry. Shown are representative staining profiles (left) and percentages of hRBCs ($CFSE^{hi}$ cells) in total $CFSE^{+}$ cells (mean ± SEM; right).

(C) Human RBCs and NOD/SCID mRBCs were labeled with CFSE, mixed at approximately 1:1 ratio, and infused into NOD/SCID mice that received repeat treatment with clodronate-liposomes (CLD, 100 μL at day −1, 50 μL at day 0, and thereafter 30 μL every 3 days; n = 3 mice

(legend continued on next page)

DISCUSSION

We have previously shown that hRBCs are rapidly rejected by recipient mouse macrophages in NOD/SCID and NSG mice (Hu et al., 2011; Hu and Yang, 2012b). Because human CD47 cross-reacts with SIRPα of the NOD background mice (Takenaka et al., 2007), the rejection of hRBCs by murine macrophages is not due to lacking of CD47-SIRPα signaling (Hu et al., 2011; Hu and Yang, 2012b). The exact mechanisms of hRBC rejection by phagocytic cells in these immunodeficient mice remain unknown.

In this study, we found that hRBCs adhere to murine F4/80^{+}Ly6G^{-} macrophages, F4/80^{-}Ly6G^{+} neutrophils, and ECs in the presence of mouse sera, indicating that both professional and non-professional phagocytic cells contribute to the clearance of hRBCs in mice. We also demonstrated that mouse complement is critical in serum-induced adherence of hRBCs to murine phagocytic cells, as the cell adherence was not observed in the presence of heated or CVF-treated mouse sera or sera from CVF-treated NOD/SCID mice. Although further studies are needed to identify the complement components that opsonize hRBCs to promote phagocytosis, the observed mouse C3 deposition on hRBCs incubated with fresh, but not heated or CVF-treated mouse sera (Figure S4), and significant C3 depletion in CVF-treated sera (Figure S3C) suggest that C3 is involved.

Immunodeficient mice on the NOD background lack hemolytic complement C5 (Baxter and Cooke, 1993), suggesting that the activity of complement in facilitating hRBC rejection is mediated by its opsonic activity. However, although the ability of mouse sera to induce hRBC adherence to murine phagocytic cells is complement dependent, hRBC survival was not improved in CVF-treated NOD/SCID mice, indicating that the rejection of hRBCs in NOD/SCID mice may occur independently of complement. However, CVF significantly prolonged the survival of hRBCs in clodronate-liposome-treated mice. We have shown that depletion of murine macrophages by clodronate-liposomes significantly prolongs hRBC survival, but is insufficient in completely preventing the rejection of hRBCs in NSG mice (Hu et al., 2011). Because injection of clodronate-liposomes efficiently depletes phagocytic macrophages, but not neutrophils or ECs (Van Rooijen and Sanders, 1994), hRBC rejection in clodronate-liposome-treated mice might reflect the ability of neutrophils and ECs to reject hRBCs. Collectively, these observations raise the possibility that complement opsonization is important for hRBC rejection by neutrophils and ECs, but is not required for hRBC rejection by phagocytic macrophages.

Complement facilitates the rejection of xenogeneic cells that are coated with xenoantigen-specific antibodies (Yang and Sykes, 2007). In this study, we found that mouse complement opsonizes hRBCs and promotes their rejection independently of antibodies. The fact that immunodeficient mice reject hRBCs but not human WBCs (Hu et al., 2011; Takenaka et al., 2007) suggests that the molecules recognized by mouse complement are likely expressed predominantly or specifically in hRBCs (Karnchanaphanurach et al., 2009; Marshall et al., 1996; Parker et al., 1984; Tambourgi et al., 2000). Identification of the molecular targets of opsonization by mouse complement would help guide the development of better methods and mouse strains for the functional characterization of hRBCs *in vivo*.

Immunodeficient mice permit human HSC engraftment and differentiation and thus offer a valuable *in vivo* model for the study of human HSC function and hematopoiesis. However, it has been difficult to study human erythropoiesis or RBC function in human HSC-grafted mice. Although there are many explanations for the lack of hRBC reconstitution in human HSC-grafted mice, we have shown that robust rejection by recipient murine macrophages is sufficient to eliminate hRBCs in these mice, wherein hRBCs become detectable following the administration of clodronate-liposomes, despite their levels remaining low (Hu et al., 2011). In this study, we show that CVF significantly improves hRBC reconstitution in HSC-grafted mice that have been depleted of macrophages by clodronate-liposomes. Thus, combination treatment with CVF and clodronate-liposomes is a simple but effective means of improving hRBC reconstitution in human HSC-grafted mice, which can be improved further by providing human cytokines that are essential for human erythropoiesis, such as interleukin-3 and erythropoietin (Chen et al., 2009; Hu et al., 2011).

EXPERIMENTAL PROCEDURES

Animals and Human Tissues and Cells

NOD.CB17-Prkdcscid/J (NOD/SCID) mice and NOD.Cg-Prkdc^{scid} B2mtm1Unc/JNju (NOD/SCID B2m) mice were purchased from Nanjing Biomedical Research Institute of Nanjing University. Animals were housed in a specific pathogen-free microisolator

per group), or with clodronate-liposomes plus CVF (CLD + CVF; 100 μL CLD plus 10 μg CVF at day −1; 50 μL CLD plus 5 μg CVF at day 0, and thereafter 30 μL CLD plus 5 μg CVF every 3 days; n = 3 mice per group). The last injection of CLD (or CLD plus CVF) was given at day 15, and the control mice were injected with PBS-liposomes (PBS; n = 2 mice per group). Shown are representative staining profiles (left; gate CFSE^{+} cells) and percentages of hRBCs (Ter119^{-} cells) in total CFSE^{+} cells (mean ± SEM; right).
*$p < 0.05$; n.s., not significant.

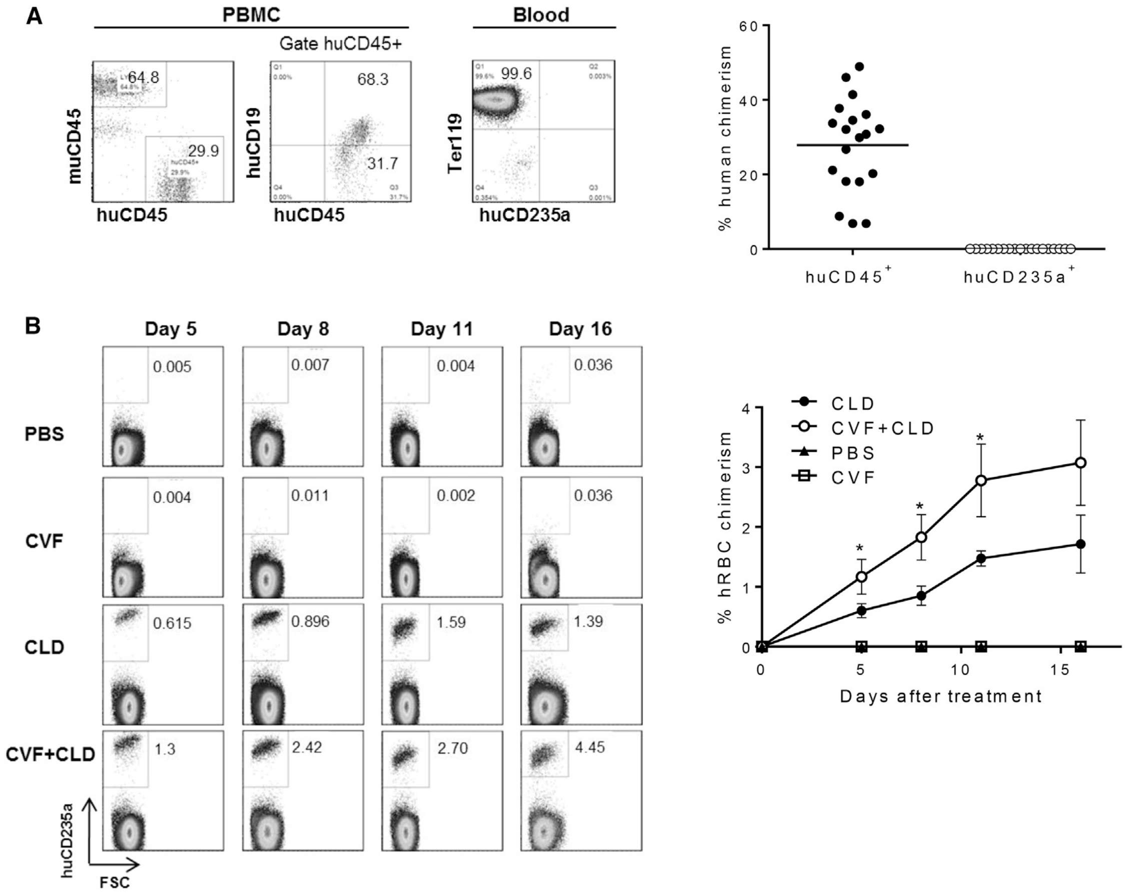

Figure 4. CVF Treatment Improves Human RBC Reconstitution in Human CD34⁺ Cell-Grafted NOD/SCID B2m Mice that Are Depleted of Macrophages

(A) Levels (%; mean ± SEM; n = 19 mice per group) of human $CD45^{+}$ cells in peripheral blood mononuclear cells (PBMCs) and of human $CD235a^{+}$ RBCs in blood at week 7 post-$CD34^{+}$ FLC injection.

(B) At week 8 post-$CD34^{+}$ FLC injection, the mice were treated with PBS (n = 3 mice per group), CLD (5 injections; 1 injection every 3 days with 100 μL for the first injection and 25 μL thereafter; n = 5 mice per group), CVF (4 injections; 5 μg/injection every 4 days starting 1 day after CLD treatment; n = 3 mice per group), or both CVF and CLD (n = 5 mice per group). Shown are levels (%; mean ± SEM) of human RBCs in blood measured by flow cytometry at the indicated time points. *$p < 0.05$.

environment and used in experiments at 5–8 weeks of age. Human blood was obtained from healthy volunteers, and human fetal liver tissues of gestational age of 17–20 weeks were obtained as discarded tissues from the First Hospital of Jilin University. Protocols involving the use of human tissues and animals were approved by the institutional review board and Institutional Animal Care and Use Committee of the First Hospital of Jilin University, and all of the experiments were performed in accordance with the protocols.

In Vitro RBC Adherence Assay

Mouse PCs collected from NOD/SCID mice 4 days after injection (i.p.) of 2% Bio-Gel polyacrylamide P100 (1 mL per mouse; Bio-Rad) were used as effector cells (Hu et al., 2011). In brief, 1×10^{6} mouse PCs were cultured at 37°C for 1 hr to allow cells to attach to the plate, and human or mouse RBCs were added, and the cells were co-cultured in 150 μL RPMI 1640 medium or NOD/SCID mouse sera for 30 min. Unbounded RBCs were removed by washing six times with PBS, and cell adherence was observed under a microscope (Olympus IX51). The area of PCs attached to the plate (including PCs without or with RBC attached) were measured by Image-Pro plus software (Paton et al., 2011), and the data are presented as adherence ratio that is calculated as: area of mouse PCs with RBC attached/area of all mouse PCs. In some experiments, magnetic-activated cell sorter (MACS)-purified mouse $F4/80^{+}$ PCs (mainly macrophages), $F4/80^{-}$ PCs (mainly neutrophils), or $CD31^{+}$ mouse ECs (isolated from NOD/SCID mouse lungs by digestion with collagenase I) were used as the effector cells in the RBC adherence assay. The sera used were either untreated (i.e., fresh), heated (at 56°C for 30 min), or complement depleted. Complement depletion was performed by addition of 5 μg CVF (Quidel) into 300 μL sera, in which PBS was used as controls.

Macrophage Depletion in Mice

Macrophage depletion *in vivo* was performed by intravenous injection of liposome-encapsulated CL2MP (clodronate-liposomes, CLD). Clodronate was purchased from Sigma, and clodronate-liposome was prepared as described (Van Rooijen and Sanders, 1994). Clodronate-liposomes were given at 100 and 50 μL per mouse at day 0 and day 1, and 30 μL per mouse, with an interval of 3 days for long-term studies. Control mice were treated on the same schedule with an equivalent volume of liposome-encapsulated PBS (PBS-liposome). The efficacy of macrophage depletion was confirmed by measuring the clearance of infused $CD47^{-/-}$ mouse RBCs in randomly selected mice as described previously (Hu et al., 2011).

Human RBC Clearance Assay

The hRBC clearance assay was performed as described previously (Hu et al., 2011). In brief, human or mouse (as control) RBCs were labeled with CFSE, mixed and injected intravenously into NOD/SCID mice (1×10^8 RBCs per mouse) that were treated with PBS-liposomes or clodronate-liposomes (100 μL at day −1, with respect to RBC transfusion), or CVF (5 μg per mouse at day −1 and day 0 before RBC transfusion). To determine the kinetics of hRBC clearance, around 5 μL blood samples were collected into heparinized tubes at various time points after transfusion and stained with anti-human CD235a or Ter119 antibodies (BD Pharmingen), and the levels of surviving transfused RBCs were measured by flow cytometric analysis.

Human $CD34^+$ Cell Transplantation in Immunodeficient Mice

NOD/SCID or NOD/SCID B2m mice were conditioned with 1.5 Gy total body irradiation and received human $CD34^+$ fetal liver cells (FLCs; $1.5–5 \times 10^5$/mouse, intravenously), as described previously (Jin et al., 2017; Lan et al., 2006). $CD34^+$ FLCs were isolated by a MACS separation system using anti-CD34 microbeads (Miltenyi Biotec). Levels of human hematopoietic cells were determined by flow cytometric analysis using various combinations of the following fluorescent-conjugated mAbs: anti-human CD45, CD19, CD235a; anti-mouse CD45 and Ter119; and isotype control mAbs (all purchased from BD Pharmingen). RBCs were collected from tail vein into the heparinized tube. Mononuclear cells were purified by density gradient centrifugation with Histopaque 1077 (Sigma-Aldrich). Analysis was performed on FACSCanto II or FACS Fortessa (BD Biosciences). Dead nucleated cells were excluded from the analysis by gating out lower forward scatter and high propidium iodide-retaining cells.

Statistical Analysis

The level of significant differences in group means was determined by the Student's t test. All statistical analysis was performed using Prism 5 (GraphPad software). A p value of ≤ 0.05 was considered significant in all analyses herein.

SUPPLEMENTAL INFORMATION

Supplemental Information includes Supplemental Experimental Procedures, four figures, and two movies and can be found with this article online at http://dx.doi.org/10.1016/j.stemcr.2017.08.018.

AUTHOR CONTRIBUTIONS

B.C., W.F., J.Z., S.-W.Z., J.H., and C.S. performed the experiments. B.C., Z.H., and Y.-G.Y. analyzed data and wrote the manuscript. T.-M.S. provided key reagents. G.-Q.Z. contributed to discussion. Z.H. and Y.-G.Y. conceived and designed the study.

ACKNOWLEDGMENTS

The authors thank Ms. Meifang Wang and Mr. Zhifu Gan for their excellent animal care. This work was supported by grants from Chinese MOST (2014AA021601, 2015CB964400, and 2013CB966900), NSFC (81570145, 81200397, 81273334, 81422026, 81571798, and 81501279), the Project of Science and Technology of Jilin Province (20140520013JH), Plan B for Bethune Project of Jilin University (101201510), the Chinese Ministry of Education (IRT1133), and the NIH (R01AI064569 and P01AI045897).

Received: January 14, 2017
Revised: August 25, 2017
Accepted: August 25, 2017
Published: September 28, 2017

REFERENCES

Baxter, A.G., and Cooke, A. (1993). Complement lytic activity has no role in the pathogenesis of autoimmune diabetes in NOD mice. Diabetes *42*, 1574–1578.

Chen, Q., Khoury, M., and Chen, J. (2009). Expression of human cytokines dramatically improves reconstitution of specific human-blood lineage cells in humanized mice. Proc. Natl. Acad. Sci. USA *106*, 21783–21788.

Da Costa, L., Galimand, J., Fenneteau, O., and Mohandas, N. (2013). Hereditary spherocytosis, elliptocytosis, and other red cell membrane disorders. Blood Rev. *27*, 167–178.

Dini, L., Lentini, A., Diez, G.D., Rocha, M., Falasca, L., Serafino, L., and Vidal-Vanaclocha, F. (1995). Phagocytosis of apoptotic bodies by liver endothelial cells. J. Cell Sci. *108* (Pt 3), 967–973.

Goodnough, L.T., Skikne, B., and Brugnara, C. (2000). Erythropoietin, iron, and erythropoiesis. Blood *96*, 823–833.

Gowda, D.C., Petrella, E.C., Raj, T.T., Bredehorst, R., and Vogel, C.W. (1994). Immunoreactivity and function of oligosaccharides in cobra venom factor. J. Immunol. *152*, 2977–2986.

Graham, A., Ford, I., Morrison, R., Barker, R.N., Greaves, M., and Erwig, L.P. (2009). Anti-endothelial antibodies interfere in apoptotic cell clearance and promote thrombosis in patients with antiphospholipid syndrome. J. Immunol. *182*, 1756–1762.

Hair, P.S., Wagner, S.M., Friederich, P.T., Drake, R.R., Nyalwidhe, J.O., and Cunnion, K.M. (2012). Complement regulator C4BP binds to *Staphylococcus aureus* and decreases opsonization. Mol. Immunol. *50*, 253–261.

Hu, Z., Van Rooijen, N., and Yang, Y.G. (2011). Macrophages prevent human red blood cell reconstitution in immunodeficient mice. Blood *118*, 5938–5946.

Hu, Z., and Yang, Y.G. (2012a). Human lymphohematopoietic reconstitution and immune function in immunodeficient mice receiving cotransplantation of human thymic tissue and CD34+ cells. Cell. Mol. Immunol. *9*, 232–236.

Hu, Z., and Yang, Y.G. (2012b). Full reconstitution of human platelets in humanized mice after macrophage depletion. Blood *120*, 1713–1716.

Jin, F., He, J., Jin, C., Fan, W., Shan, Y., Zhang, Z., Sun, L., Hu, Z., and Yang, Y.G. (2017). Antithymocyte globulin treatment at the time of transplantation impairs donor hematopoietic stem cell engraftment. Cell. Mol. Immunol. *14*, 443–450.

Karnchanaphanurach, P., Mirchev, R., Ghiran, I., Asara, J.M., Papahadjopoulos-Sternberg, B., Nicholson-Weller, A., and Golan, D.E. (2009). C3b deposition on human erythrocytes induces the formation of a membrane skeleton-linked protein complex. J. Clin. Invest. *119*, 788–801.

Lan, P., Tonomura, N., Shimizu, A., Wang, S., and Yang, Y.G. (2006). Reconstitution of a functional human immune system in immunodeficient mice through combined human fetal thymus/liver and CD34+ cell transplantation. Blood *108*, 487–492.

Lewis, L.A., Ram, S., Prasad, A., Gulati, S., Getzlaff, S., Blom, A.M., Vogel, U., and Rice, P.A. (2008). Defining targets for complement components C4b and C3b on the pathogenic neisseriae. Infect. Immun. *76*, 339–350.

Marshall, P., Hasegawa, A., Davidson, E.A., Nussenzweig, V., and Whitlow, M. (1996). Interaction between complement proteins C5b-7 and erythrocyte membrane sialic acid. J. Exp. Med. *184*, 1225–1232.

Nauta, A.J., Castellano, G., Xu, W., Woltman, A.M., Borrias, M.C., Daha, M.R., van Kooten, C., and Roos, A. (2004). Opsonization with C1q and mannose-binding lectin targets apoptotic cells to dendritic cells. J. Immunol. *173*, 3044–3050.

Palis, J. (2014). Primitive and definitive erythropoiesis in mammals. Front. Physiol. *5*, 3.

Parker, C.J., Soldato, C.M., and Telen, M.J. (1984). Increased efficiency of binding of nascent C3b to the erythrocytes of chronic cold agglutinin disease. J. Clin. Invest. *74*, 1050–1062.

Patel, P.C., and Harrison, R.E. (2008). Membrane ruffles capture C3bi-opsonized particles in activated macrophages. Mol. Biol. Cell *19*, 4628–4639.

Paton, D., Faragher, B., Mustaffa, K.M., Szestak, T., Barrett, S.D., and Craig, A.G. (2011). Automated counting for *Plasmodium falciparum* cytoadherence experiments. Malar. J. *10*, 91.

Rahmig, S., Kronstein-Wiedemann, R., Fohgrub, J., Kronstein, N., Nevmerzhitskaya, A., Bornhauser, M., Gassmann, M., Platz, A., Ordemann, R., Tonn, T., et al. (2016). Improved human erythropoiesis and platelet formation in humanized NSGW41 mice. Stem Cell Reports *7*, 591–601.

Rees, D.C., Williams, T.N., and Gladwin, M.T. (2010). Sickle-cell disease. Lancet *376*, 2018–2031.

Rongvaux, A., Takizawa, H., Strowig, T., Willinger, T., Eynon, E.E., Flavell, R.A., and Manz, M.G. (2013). Human hemato-lymphoid system mice: current use and future potential for medicine. Annu. Rev. Immunol. *31*, 635–674.

Sankaran, V.G., and Weiss, M.J. (2015). Anemia: progress in molecular mechanisms and therapies. Nat. Med. *21*, 221–230.

Suzuki, K., Tsunekawa, Y., Hernandez-Benitez, R., Wu, J., Zhu, J., Kim, E.J., Hatanaka, F., Yamamoto, M., Araoka, T., Li, Z., et al. (2016). In vivo genome editing via CRISPR/Cas9 mediated homology-independent targeted integration. Nature *540*, 144–149.

Takenaka, K., Prasolava, T.K., Wang, J.C., Mortin-Toth, S.M., Khalouei, S., Gan, O.I., Dick, J.E., and Danska, J.S. (2007). Polymorphism in Sirpa modulates engraftment of human hematopoietic stem cells. Nat. Immunol. *8*, 1313–1323.

Tambourgi, D.V., Morgan, B.P., de Andrade, R.M., Magnoli, F.C., and van Den Berg, C.W. (2000). Loxosceles intermedia spider envenomation induces activation of an endogenous metalloproteinase, resulting in cleavage of glycophorins from the erythrocyte surface and facilitating complement-mediated lysis. Blood *95*, 683–691.

Van Rooijen, N., and Sanders, A. (1994). Liposome mediated depletion of macrophages: mechanism of action, preparation of liposomes and applications. J. Immunol. Methods *174*, 83–93.

Yang, Y.G., and Sykes, M. (2007). Xenotransplantation: current status and a perspective on the future. Nat. Rev. Immunol. *7*, 519–531.

Yu, Z., Chen, T., and Cao, X. (2016). Neutrophil sensing of cytoplasmic, pathogenic DNA in a cGAS-STING-independent manner. Cell. Mol. Immunol. *13*, 411–414.

Stem Cell Reports, Volume *9*

Supplemental Information

Complement Depletion Improves Human Red Blood Cell Reconstitution in Immunodeficient Mice

Bing Chen, Wei Fan, Jun Zou, Siwen Zhang, Jin He, Chang Shu, Guoqing Zhao, Tianmeng Sun, Zheng Hu, and Yong-Guang Yang

Supplemental Information

Complement depletion improves human red blood cell reconstitution in immunodeficient mice

Bing Chen, Wei Fan, Jun Zou, Siwen Zhang, Jin He, Chang Shu, Guoqing Zhao, Tianmeng Sun, Zheng Hu, Yong-Guang Yang

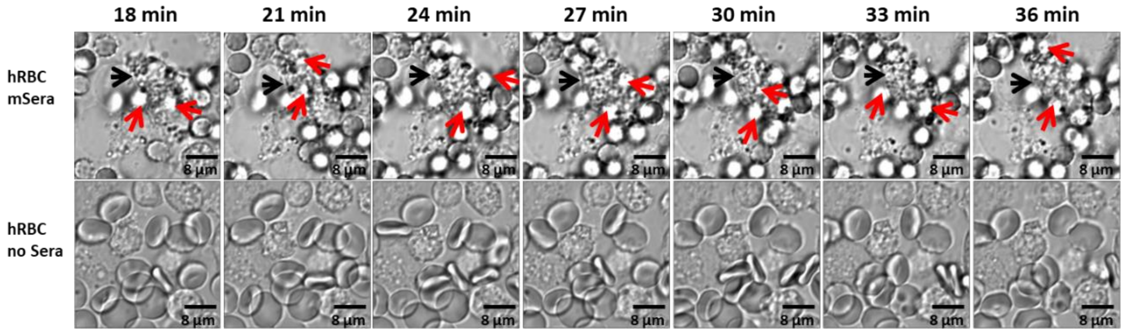

Figure S1. Human RBC adherence to and engulfment by mouse phagocytic cells.
Human RBCs were co-cultured with NOD/SCID mouse peritoneal cells in the presence of 15% NOD/SCID mouse sera (top) or absence of sera (bottom) at 37 °C in Delta Vision Imaging System (the same experiment shown in Movie S1 and S2). Shown are representative imagines captured at indicated time points. In the cultures with mouse sera (top), human RBCs and mouse phagocytic cells are distinguished by red and black arrows, respectively. Scale bars represent 8 µm.

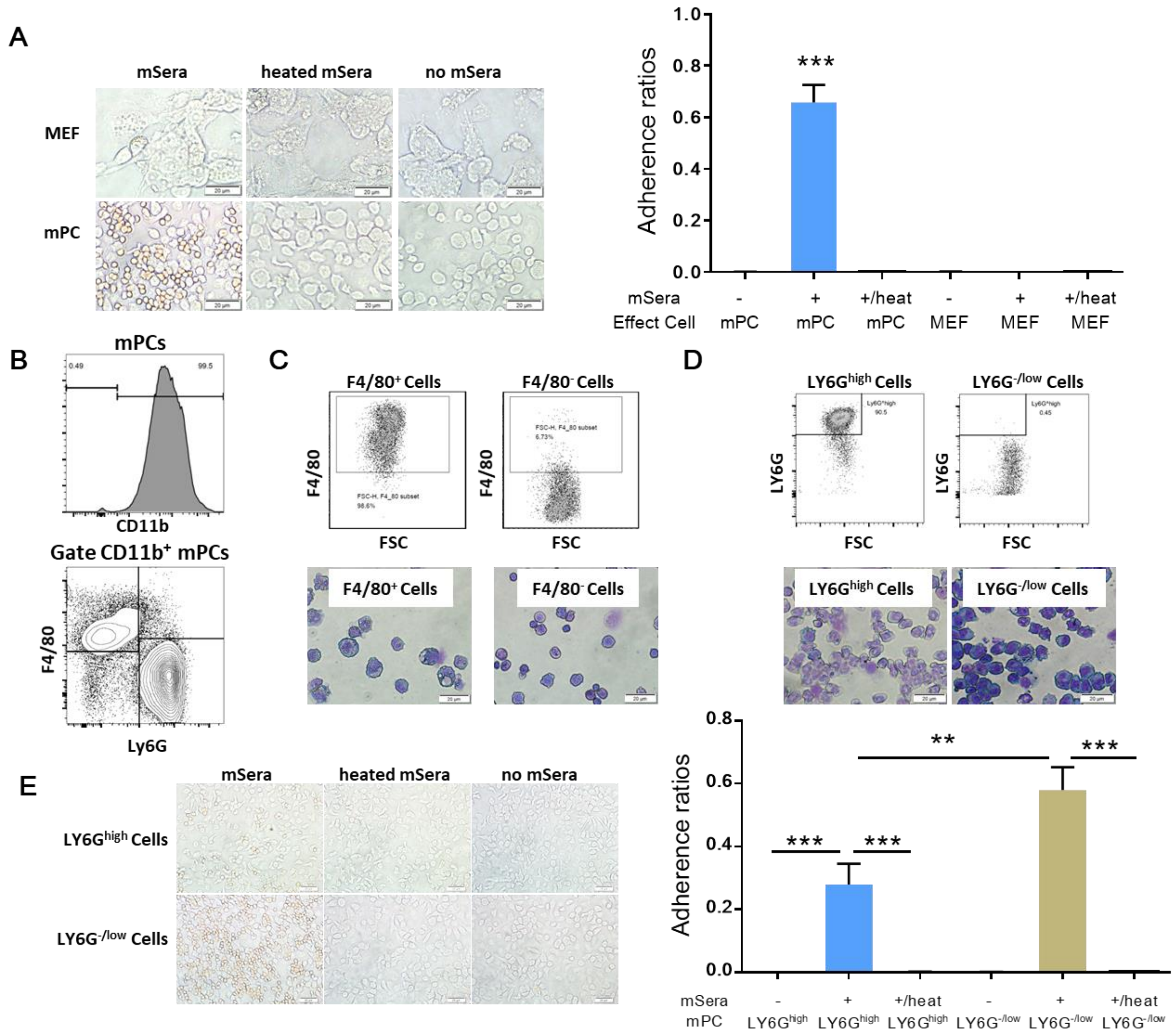

Figure S2. Adherence of human RBCs to mouse phagocytic and non-phagocytic cells.
(A) Adherence of human RBCs (hRBCs) to NOD/SCID mouse peritoneal cells (mPCs) or mouse embryonic fibroblast (MEF) cells in the presence or absence of NOD/SCID mouse sera (mSera). Representative pictures (left) and adherence ratios (right; mean±SDs; n=4 technical replicates) are shown. ***, p<0.001. (B) Flow cytometric analysis of CD11b, F4/80 and Ly6G expression on mPCs. (C, D) F4/80$^+$ and F4/80$^-$ (C) or LY6G$^+$ and LY6G$^-$ (D) mPCs were isolated by magnetic-activated cell sorting, and stained with Wright-Giemsa staining after cytospin. Flow cytometry profiles showing the purity of the sorted cells (top) and representative images of Wright-Giemsa staining (bottom) are shown. (E) Adherence of hRBCs to purified LY6G$^+$ and LY6G$^-$ mPCs in the presence of untreated or heated NOD/SCID mouse sera (mSera), or in serum-free medium. Representative pictures (left) and adherence ratios (right; mean±SDs; n=4 technical replicates) are shown. Scale bars represent 20 μm. **, p<0.01; ***, p<0.001.

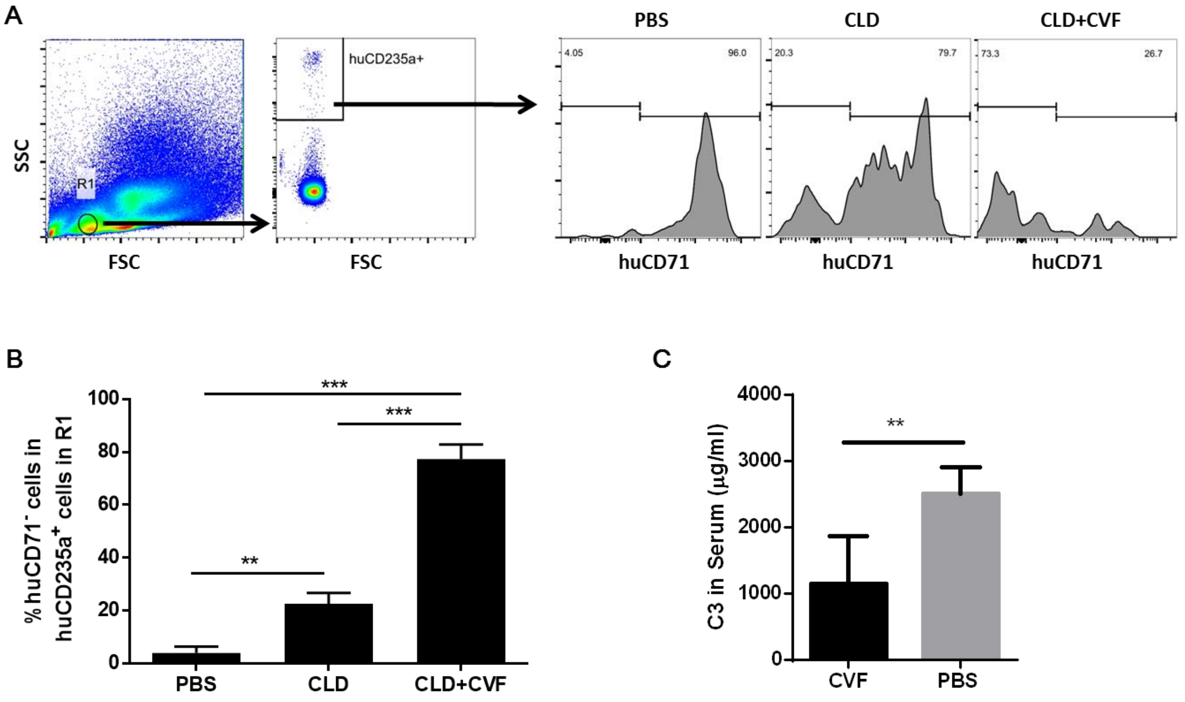

Figure S3. CVF treatment improves human erythropoiesis in human CD34^{+} cell-grafted NOD/SCID mice that are depleted of macrophages.

NOD/SCID mice were irradiated and injected with 3×10^{5} CD34^{+} cells, and treated 4 weeks later with PBS (n=3 mice per group), clodronate-liposomes (CLD; 3 injections; 100 µL at day 0, 25 µL at day 2 and day 5; n=3 mice per group), or CLD and CVF (3 injections; 5 µg at day 0, 2, 5; n=3 mice per group). Animals were sacrificed 7 days later and analyzed for human CD71^{-}CD235a^{+} erythroid cells in bone marrow by flow cytometry. (A) Representative staining profiles of human CD71 expression on gated human CD235a^{+} cells in the "mature erythrocyte–enriched" gate (R1). (B) Percentages (mean±SDs) of CD71^{-}CD235a^{+} erythroid cells in R1 gate. (C) Levels of mouse serum C3 in CVF-treated (n=3 mice per group) and PBS-injected (n=6 mice per group) mice at day 7 after treatment. **, $p<0.01$; ***, $p<0.001$.

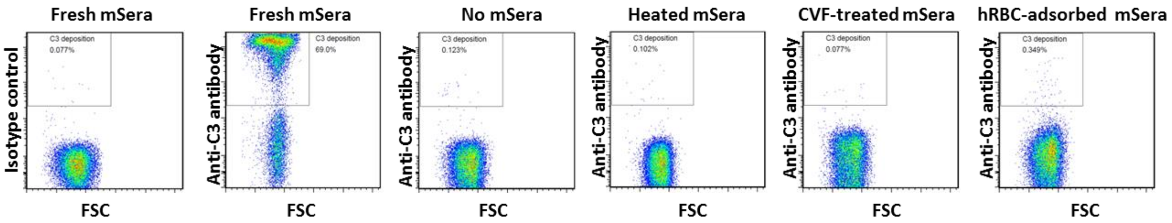

Figure S4. Mouse complement deposition on human RBCs opsonized with mouse sera. Human RBCs were incubated in fresh, heated, CVF treated, or human RBC-adsorbed mouse sera (mSera), or in serum-free medium for 30 minutes, and analyzed for C3 deposition by flow cytometry. Representative flow cytometric profiles from a representative of 2 independent experiments are shown.

Supplemental Movies

Movie S1. Human RBCs and mouse PCs cultured in the presence of 15% NOD/SCID mouse sera.
Movie S2. Human RBCs and mouse PCs cultured in absence of mouse sera.

Supplemental Experimental Procedures

Morphologic analyses of mouse peritoneal cavity cells from NOD/SCID mice

F4/80$^+$ and F4/80$^-$ or LY6G$^+$ and LY6G$^-$ mouse PCs were purified from NOD/SCID mouse peritoneal cavity 4 days after Bio-Gel i.p. injection, suspended in PBS, and centrifuged (130g for 5 minutes) onto glass slides using a Cytospin centrifuge (Shandon). The slides were stained with Wright Giemsa staining kit (Leagene). Stained slides were examined under an Olympus microscope and photographed using an Olympus DP73 digital color camera.

ELISA

Concentration of mouse complement C3 in mouse sera was analyzed by ELISA kit specific for mouse complement C3 (ab157711, Abcam), according to the manufacture's instructions.

Complement deposition assay

Human RBCs were incubated for 30 minutes in serum-free medium, or with fresh, heated, CVF-treated (at 5μg CVF per 300 μL sera), or human RBC-adsorbed (incubated with hRBCs at 5×10^9 cells per 300 μL sera) NOD/SCID mouse sera. After incubation, the cells were washed twice and stained with rat anti-mouse complement C3 mAb (CL7503, CEDARLANE) or rat IgG_{2a} isotype control antibody, and analyzed by flow cytometry.
